# Hexane-1,6-di­ammonium hexa­fluoro­silicate

**DOI:** 10.1107/S1600536813034144

**Published:** 2013-12-24

**Authors:** Ali Ouasri, Ali Rhandour, Mohamed Saadi, Lahcen El Ammari

**Affiliations:** a Département de Physique-Chimie, Laboratoire de Chimie, Centre Régional des Métiers de l’Education et de la Formation, Souissi Rabat, Morocco; b Equipe de Physico-Chimie des Matériaux Inorganiques, Université Ibn Tofail, Faculté des Sciences, BP 133, 14000 Kénitra, Morocco; c Laboratoire de Chimie du Solide Appliquée, Faculté des Sciences, Université Mohammed V-Agdal, Avenue Ibn Battouta, BP 1014, Rabat, Morocco

## Abstract

The asymmetric unit of the title organic–inorganic molecular salt, C_6_H_18_N_2_^2+^·SiF_6_^2−^, consists of one anion and one cation together with half of each of two cations and two anions located on inversion centres. The SiF_6_^2−^ octa­hedral anions are arranged to form sheets parallel to (011), which are linked into a three-dimensional network by the organic cations through N—H⋯F hydrogen bonds.

## Related literature   

For background to potential physical properties of alkyl­diammonium halogenometallate salts, see: Ouasri *et al.* (2003 ▶); Elyoubi *et al.* (2004 ▶). For the structures of related compounds, see: Jeghnou *et al.* (2005 ▶); Ouasri *et al.* (2012 ▶, 2013*a* ▶,*b* ▶); Rhandour *et al.* (2011 ▶); Elaoud *et al.* (1995 ▶).
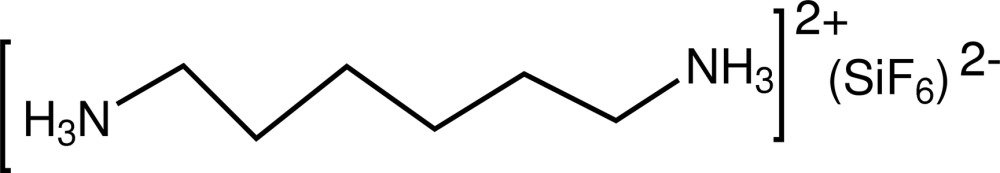


## Experimental   

### 

#### Crystal data  

C_6_H_18_N_2_^2+^·SiF_6_^2−^
*M* *_r_* = 260.31
Triclinic, 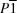

*a* = 5.8965 (2) Å
*b* = 13.6946 (5) Å
*c* = 14.4945 (5) Å
α = 91.379 (2)°
β = 92.797 (2)°
γ = 90.906 (2)°
*V* = 1168.53 (7) Å^3^
*Z* = 4
Mo *K*α radiation
μ = 0.25 mm^−1^
*T* = 296 K
0.37 × 0.33 × 0.28 mm

#### Data collection  

Bruker X8 APEX diffractometer
Absorption correction: multi-scan (*SADABS*; Bruker, 2009 ▶) *T*_min_ = 0.686, *T*_max_ = 0.747
29127 measured reflections
4760 independent reflections
3618 reflections with *I* > 2σ(*I*)
*R*_int_ = 0.023

#### Refinement  

*R*[*F* ^2^ > 2σ(*F* ^2^)] = 0.052
*wR*(*F* ^2^) = 0.159
*S* = 1.06
4760 reflections
274 parameters
H-atom parameters constrained
Δρ_max_ = 0.46 e Å^−3^
Δρ_min_ = −0.26 e Å^−3^

### 

Data collection: *APEX2* (Bruker, 2009 ▶); cell refinement: *SAINT-Plus* (Bruker, 2009 ▶); data reduction: *SAINT-Plus*; program(s) used to solve structure: *SHELXS97* (Sheldrick, 2008 ▶); program(s) used to refine structure: *SHELXL97* (Sheldrick, 2008 ▶); molecular graphics: *ORTEP-3 for Windows* (Farrugia, 2012 ▶); software used to prepare material for publication: *PLATON* (Spek, 2009 ▶) and *publCIF* (Westrip, 2010 ▶).

## Supplementary Material

Crystal structure: contains datablock(s) I. DOI: 10.1107/S1600536813034144/rz5101sup1.cif

Structure factors: contains datablock(s) I. DOI: 10.1107/S1600536813034144/rz5101Isup2.hkl

Additional supporting information:  crystallographic information; 3D view; checkCIF report

## Figures and Tables

**Table 1:** Hydrogen-bond geometry (Å, °)

*D*—H⋯*A*	*D*—H	H⋯*A*	*D*⋯*A*	*D*—H⋯*A*
N2—H2*NC*⋯F5	0.89	2.31	3.062 (3)	142
N2—H2*NC*⋯F6	0.89	2.27	3.063 (3)	148
N2—H2*NB*⋯F7	0.89	2.52	3.095 (3)	123
N2—H2*NB*⋯F8	0.89	2.03	2.917 (3)	175
N3—H3*NA*⋯F1	0.89	2.06	2.934 (3)	169
N4—H4*NA*⋯F8	0.89	2.34	3.095 (3)	143
N4—H4*NA*⋯F9	0.89	2.14	2.923 (3)	146
N4—H4*NC*⋯F6	0.89	2.00	2.885 (3)	172
N1—H1*NB*⋯F11^i^	0.89	2.07	2.895 (3)	155
N1—H1*NA*⋯F10^iii^	0.89	2.01	2.869 (3)	163
N1—H1*NC*⋯F2^ii^	0.89	2.02	2.857 (2)	155
N2—H2*NA*⋯F7^iv^	0.89	2.02	2.906 (3)	170
N3—H3*NB*⋯F10^v^	0.89	2.48	3.239 (3)	144
N3—H3*NB*⋯F12	0.89	2.13	2.907 (2)	145
N3—H3*NC*⋯F3^vi^	0.89	2.02	2.904 (3)	170
N3—H3*NC*⋯F4^vi^	0.89	2.44	3.034 (3)	124
N4—H4*NB*⋯F4^vi^	0.89	2.01	2.832 (3)	154
N4—H4*NB*⋯F5^vi^	0.89	2.51	3.086 (3)	123

Symmetry codes: (i) 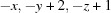; (ii) 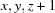; (iii) 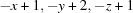; (iv) 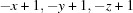; (v) 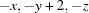; (vi) 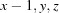.

**Fig. 1.**
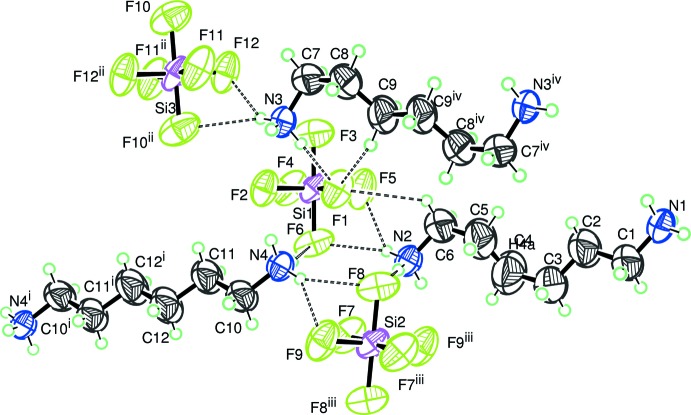
The molecular structure of the title compound with displacement ellipsoids drawn at the 50% probability level. H atoms are represented as small circles. Symmetry codes: (i) -x, 1.-y, -z; (ii) -x, 2.-y, -z; (iii) -x, 1.-y, 1.-z; (iv) -x, 2.-y, 1.-z.

**Fig. 2.**
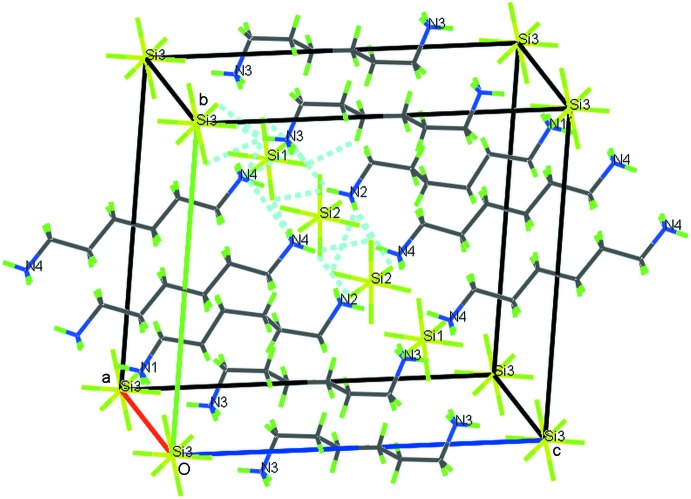
Packing diagram of the title compound, showing inorganic layers linked through N–H···F hydrogen bonds (dashed lines).

## supplementary crystallographic information

### 1. Comment

The title compound belongs to the alkyldiammonium halogenometallate salts family of general formula (NH_3_(CH_2_)_n_NH_3_)*MX*_6_ where *M* is Sn, Si, Te and *X* is Cl, Br, I and F. These compounds have recently attracted the interest of many investigators due to their potential physical properties (Ouasri *et al.*, 2003; Elyoubi *et al.*, 2004). X-ray, thermal and vibrational studies of phase transitions have been performed for others compounds which belong to the alkyldiammonium halogenobismuthate salts family such as the pentachlorobismuthate derivative (NH_3_(CH_2_)_n_NH_3_)BiCl_5_ (Jeghnou *et al.*, 2005; Ouasri *et al.*, 2012; Rhandour *et al.*, 2011; Ouasri *et al.*, 2013*a*; Ouasri *et al.*, 2013*b*). It was found that hexahalogenometallates (NH_3_(CH_2_)_n_NH_3_)*MX*_6_ (where *M*: Sn, Te; *X*: Cl, Br, I) have been more studied and evoked that their hexafluorosilicate homologous, whose (NH_3_(CH_2_)_6_NH_3_)SiF_6_ (Elaoud *et al.*, 1995) is the only compound known to date. The aim of the present paper was to study the structure of the recently synthesized hexyldiammonium hexafluorosilicate (NH_3_(CH_2_)_6_NH_3_)SiF_6_ crystals by X-ray diffraction at room temperature.

The structure of the title compound is built up from inorganic anions linked to organic cations through hydrogen bonds as shown in Fig. 1. In this structure, all atoms are in general positions, except two silicon atoms [Si2 (0, 1/2, 1/2); Si3 (0, 0, 0)] located at inversion centres of the *P*1 space group. Moreover, the unit cell contains one organic cation and two halves of cations located about an inversion centre. Each silicon atom is surrounded by six fluorine anions in a slightly distorted SiF_6_^2-^ octahedral geometry. The SiF_6_^2-^ octahedra form two-dimensional layers parallel to the (0 1 1) plane. The hexanediammonium cations fill the space between the inorganic sheets, forming a three-dimensional network by N–H···F hydrogen bonds (Fig. 2; Table 1).

### 2. Experimental

Single crystals of the title compound were obtained by slow evaporation at room temperature of an aqueous solution containing stoichiometric amounts of 1,6-hexanediamine NH_2_(CH_2_)_6_NH_2_ and hexafluorosilicic acid H_2_SiF_6_.

### 3. Refinement

H atoms were located in a difference Fourier map and treated as riding, with C—H = 0.97 Å, N—H = 0.89 Å, and with *U*_iso_(H) = 1.2 *U*_eq_ (C, N). Four outlier (0 1 0, 0 0 1, 0 1 1, 1 -1 1) were omitted in the last refinement cycles.

### Crystal data

**Table:**

C_6_H_18_N_2_^2+^·SiF_6_^2^^−^	*Z* = 4
*M**_r_* = 260.31	*F*(000) = 544
Triclinic, *P*1	*D*_x_ = 1.480 Mg m^−^^3^
*a* = 5.8965 (2) Å	Mo *K*α radiation, λ = 0.71073 Å
*b* = 13.6946 (5) Å	Cell parameters from 4760 reflections
*c* = 14.4945 (5) Å	θ = 2.0–26.4°
α = 91.379 (2)°	µ = 0.25 mm^−^^1^
β = 92.797 (2)°	*T* = 296 K
γ = 90.906 (2)°	Block, colourless
*V* = 1168.53 (7) Å^3^	0.37 × 0.33 × 0.28 mm

### Data collection

**Table:**

Bruker X8 APEX diffractometer	4760 independent reflections
Radiation source: fine-focus sealed tube	3618 reflections with *I* > 2σ(*I*)
Graphite monochromator	*R*_int_ = 0.023
φ and ω scans	θ_max_ = 26.4°, θ_min_ = 2.0°
Absorption correction: multi-scan (*SADABS*; Bruker, 2009)	*h* = −7→7
*T*_min_ = 0.686, *T*_max_ = 0.747	*k* = −17→17
29127 measured reflections	*l* = −18→18

### Refinement

**Table:**

Refinement on *F*^2^	Primary atom site location: structure-invariant direct methods
Least-squares matrix: full	Secondary atom site location: difference Fourier map
*R*[*F*^2^ > 2σ(*F*^2^)] = 0.052	Hydrogen site location: inferred from neighbouring sites
*wR*(*F*^2^) = 0.159	H-atom parameters constrained
*S* = 1.06	*w* = 1/[σ^2^(*F*_o_^2^) + (0.0734*P*)^2^ + 1.0436*P*] where *P* = (*F*_o_^2^ + 2*F*_c_^2^)/3
4760 reflections	(Δ/σ)_max_ < 0.001
274 parameters	Δρ_max_ = 0.46 e Å^−^^3^
0 restraints	Δρ_min_ = −0.26 e Å^−^^3^

### Special details

**Table:**

Geometry. All e.s.d.'s (except the e.s.d. in the dihedral angle between two l.s. planes) are estimated using the full covariance matrix. The cell e.s.d.'s are taken into account individually in the estimation of e.s.d.'s in distances, angles and torsion angles; correlations between e.s.d.'s in cell parameters are only used when they are defined by crystal symmetry. An approximate (isotropic) treatment of cell e.s.d.'s is used for estimating e.s.d.'s involving l.s. planes.
Refinement. Refinement of *F*^2^ against all reflections. The weighted *R*-factor *wR* and goodness of fit *S* are based on *F*^2^, conventional *R*-factors *R* are based on *F*, with *F* set to zero for negative *F*^2^. The threshold expression of *F*^2^ > σ(*F*^2^) is used only for calculating *R*-factors(gt) *etc*. and is not relevant to the choice of reflections for refinement. *R*-factors based on *F*^2^ are statistically about twice as large as those based on *F*, and *R*- factors based on all data will be even larger.

### Fractional atomic coordinates and isotropic or equivalent isotropic displacement parameters (Å^2^)

**Table:**

	*x*	*y*	*z*	*U*_iso_*/*U*_eq_	
C1	0.4600 (7)	0.7541 (2)	0.9702 (2)	0.0601 (9)	
H1A	0.2997	0.7367	0.9682	0.072*	
H1B	0.5425	0.7018	1.0000	0.072*	
C2	0.5353 (7)	0.7632 (3)	0.8734 (2)	0.0632 (9)	
H2A	0.4561	0.8168	0.8443	0.076*	
H2B	0.6965	0.7788	0.8755	0.076*	
C3	0.4917 (8)	0.6720 (3)	0.8158 (2)	0.0773 (12)	
H3A	0.3295	0.6585	0.8117	0.093*	
H3B	0.5638	0.6180	0.8471	0.093*	
C4	0.5760 (8)	0.6754 (3)	0.7179 (2)	0.0828 (12)	
H4A	0.7366	0.6925	0.7221	0.099*	
H4B	0.5608	0.6103	0.6902	0.099*	
C5	0.4604 (8)	0.7437 (3)	0.6558 (2)	0.0739 (11)	
H5A	0.4718	0.8083	0.6848	0.089*	
H5B	0.3006	0.7254	0.6507	0.089*	
C6	0.5433 (7)	0.7508 (2)	0.5600 (2)	0.0624 (9)	
H6A	0.4661	0.8039	0.5292	0.075*	
H6B	0.7043	0.7669	0.5642	0.075*	
C7	0.0516 (6)	1.0370 (2)	0.2857 (2)	0.0523 (7)	
H7A	−0.0151	1.0834	0.2432	0.063*	
H7B	0.2133	1.0513	0.2925	0.063*	
C8	−0.0506 (6)	1.0498 (2)	0.3775 (2)	0.0552 (8)	
H8A	−0.2110	1.0330	0.3703	0.066*	
H8B	−0.0383	1.1184	0.3959	0.066*	
C9	0.0530 (6)	0.9906 (2)	0.45450 (19)	0.0552 (8)	
H9A	0.0358	0.9217	0.4379	0.066*	
H9B	0.2143	1.0058	0.4614	0.066*	
C10	−0.0545 (6)	0.5465 (2)	0.21254 (19)	0.0497 (7)	
H10A	−0.2183	0.5377	0.2075	0.060*	
H10B	0.0104	0.4860	0.2339	0.060*	
C11	0.0292 (6)	0.5675 (2)	0.11971 (19)	0.0524 (7)	
H11A	0.1936	0.5724	0.1243	0.063*	
H11B	−0.0279	0.6302	0.1003	0.063*	
C12	−0.0417 (7)	0.4908 (3)	0.0471 (2)	0.0617 (9)	
H12A	0.0139	0.4281	0.0672	0.074*	
H12B	−0.2062	0.4864	0.0426	0.074*	
N1	0.4970 (3)	0.84453 (16)	1.02554 (13)	0.0367 (5)	
H1NB	0.4442	0.8948	0.9937	0.044*	
H1NA	0.6449	0.8535	1.0388	0.044*	
H1NC	0.4243	0.8402	1.0777	0.044*	
N2	0.5073 (3)	0.66128 (16)	0.50333 (14)	0.0357 (5)	
H2NA	0.5824	0.6126	0.5298	0.043*	
H2NC	0.5580	0.6706	0.4473	0.043*	
H2NB	0.3597	0.6463	0.4985	0.043*	
N3	0.0159 (3)	0.93712 (15)	0.24637 (13)	0.0341 (5)	
H3NA	0.0877	0.8948	0.2826	0.041*	
H3NB	0.0703	0.9338	0.1902	0.041*	
H3NC	−0.1320	0.9226	0.2426	0.041*	
N4	0.0039 (3)	0.62479 (15)	0.28063 (13)	0.0326 (4)	
H4NA	−0.0539	0.6104	0.3343	0.039*	
H4NB	−0.0532	0.6808	0.2609	0.039*	
H4NC	0.1542	0.6308	0.2880	0.039*	
F1	0.3057 (2)	0.80063 (13)	0.34886 (10)	0.0471 (4)	
F2	0.3060 (2)	0.77137 (12)	0.18810 (10)	0.0437 (4)	
F3	0.5306 (3)	0.90186 (12)	0.25488 (13)	0.0553 (5)	
F4	0.7096 (2)	0.76175 (13)	0.19650 (11)	0.0476 (4)	
F5	0.7122 (3)	0.79006 (15)	0.35786 (11)	0.0560 (5)	
F6	0.4882 (3)	0.66026 (12)	0.29172 (12)	0.0496 (4)	
F7	0.2030 (2)	0.49066 (13)	0.42253 (11)	0.0483 (4)	
F8	0.0188 (3)	0.62264 (11)	0.49428 (12)	0.0516 (4)	
F9	−0.2025 (3)	0.49935 (13)	0.41489 (11)	0.0514 (4)	
F10	0.0226 (3)	1.11843 (13)	−0.02764 (13)	0.0575 (5)	
F11	−0.1963 (2)	1.02734 (14)	0.07661 (10)	0.0519 (5)	
F12	0.2087 (2)	1.01509 (14)	0.08206 (10)	0.0505 (4)	
Si1	0.50922 (10)	0.78157 (5)	0.27367 (5)	0.0328 (2)	
Si2	0.0000	0.5000	0.5000	0.0339 (2)	
Si3	0.0000	1.0000	0.0000	0.0370 (3)	

### Atomic displacement parameters (Å^2^)

**Table:**

	*U*^11^	*U*^22^	*U*^33^	*U*^12^	*U*^13^	*U*^23^
C1	0.084 (2)	0.0542 (19)	0.0415 (16)	−0.0097 (17)	0.0083 (15)	−0.0045 (14)
C2	0.081 (2)	0.064 (2)	0.0448 (17)	−0.0093 (18)	0.0126 (16)	−0.0058 (15)
C3	0.128 (4)	0.057 (2)	0.0463 (19)	−0.002 (2)	0.007 (2)	−0.0031 (16)
C4	0.118 (3)	0.081 (3)	0.050 (2)	0.025 (2)	0.005 (2)	−0.0012 (19)
C5	0.105 (3)	0.067 (2)	0.0488 (19)	0.016 (2)	−0.0007 (19)	−0.0104 (17)
C6	0.092 (3)	0.0506 (19)	0.0448 (17)	−0.0089 (17)	0.0047 (16)	−0.0023 (14)
C7	0.073 (2)	0.0411 (16)	0.0428 (16)	−0.0057 (14)	0.0037 (14)	0.0020 (12)
C8	0.076 (2)	0.0458 (17)	0.0431 (16)	0.0089 (15)	0.0005 (14)	−0.0075 (13)
C9	0.074 (2)	0.0551 (19)	0.0367 (15)	0.0095 (16)	0.0021 (14)	−0.0084 (13)
C10	0.0667 (19)	0.0424 (16)	0.0400 (15)	−0.0072 (14)	0.0086 (13)	−0.0043 (12)
C11	0.068 (2)	0.0513 (18)	0.0378 (15)	−0.0085 (15)	0.0098 (13)	−0.0074 (13)
C12	0.085 (2)	0.060 (2)	0.0407 (17)	−0.0171 (18)	0.0137 (16)	−0.0115 (14)
N1	0.0343 (11)	0.0477 (13)	0.0284 (10)	−0.0005 (9)	0.0031 (8)	0.0055 (9)
N2	0.0327 (11)	0.0439 (12)	0.0312 (10)	0.0027 (9)	0.0046 (8)	0.0050 (9)
N3	0.0349 (11)	0.0409 (12)	0.0267 (10)	0.0020 (9)	0.0030 (8)	0.0023 (8)
N4	0.0320 (10)	0.0375 (11)	0.0289 (10)	0.0021 (8)	0.0038 (8)	0.0032 (8)
F1	0.0376 (8)	0.0681 (11)	0.0373 (8)	0.0117 (7)	0.0124 (6)	0.0080 (7)
F2	0.0325 (8)	0.0626 (10)	0.0359 (8)	0.0011 (7)	−0.0024 (6)	0.0082 (7)
F3	0.0435 (9)	0.0435 (9)	0.0799 (12)	0.0011 (7)	0.0045 (8)	0.0161 (8)
F4	0.0340 (8)	0.0681 (11)	0.0431 (9)	0.0119 (7)	0.0149 (6)	0.0189 (8)
F5	0.0363 (8)	0.0857 (13)	0.0454 (9)	0.0008 (8)	−0.0084 (7)	0.0099 (9)
F6	0.0425 (9)	0.0421 (9)	0.0662 (11)	0.0062 (7)	0.0118 (7)	0.0212 (8)
F7	0.0371 (8)	0.0635 (11)	0.0468 (9)	0.0107 (7)	0.0178 (7)	0.0171 (8)
F8	0.0465 (9)	0.0389 (9)	0.0708 (11)	0.0031 (7)	0.0104 (8)	0.0149 (8)
F9	0.0358 (8)	0.0743 (12)	0.0443 (9)	−0.0001 (8)	−0.0040 (7)	0.0178 (8)
F10	0.0428 (9)	0.0582 (11)	0.0726 (12)	−0.0008 (8)	0.0034 (8)	0.0268 (9)
F11	0.0351 (8)	0.0858 (13)	0.0366 (8)	0.0119 (8)	0.0090 (6)	0.0158 (8)
F12	0.0311 (8)	0.0823 (13)	0.0380 (8)	−0.0017 (8)	−0.0052 (6)	0.0121 (8)
Si1	0.0246 (3)	0.0431 (4)	0.0318 (4)	0.0035 (3)	0.0040 (3)	0.0126 (3)
Si2	0.0250 (5)	0.0411 (6)	0.0368 (5)	0.0039 (4)	0.0067 (4)	0.0142 (4)
Si3	0.0244 (5)	0.0575 (7)	0.0298 (5)	0.0011 (4)	0.0022 (4)	0.0166 (4)

### Geometric parameters (Å, º)

**Table:**

C1—N1	1.466 (4)	C11—H11B	0.9700
C1—C2	1.500 (4)	C12—C12^ii^	1.499 (6)
C1—H1A	0.9700	C12—H12A	0.9700
C1—H1B	0.9700	C12—H12B	0.9700
C2—C3	1.498 (5)	N1—H1NB	0.8900
C2—H2A	0.9700	N1—H1NA	0.8900
C2—H2B	0.9700	N1—H1NC	0.8900
C3—C4	1.527 (5)	N2—H2NA	0.8900
C3—H3A	0.9700	N2—H2NC	0.8900
C3—H3B	0.9700	N2—H2NB	0.8900
C4—C5	1.467 (5)	N3—H3NA	0.8900
C4—H4A	0.9700	N3—H3NB	0.8900
C4—H4B	0.9700	N3—H3NC	0.8900
C5—C6	1.499 (5)	N4—H4NA	0.8900
C5—H5A	0.9700	N4—H4NB	0.8900
C5—H5B	0.9700	N4—H4NC	0.8900
C6—N2	1.466 (4)	F1—Si1	1.6794 (15)
C6—H6A	0.9700	F2—Si1	1.6837 (15)
C6—H6B	0.9700	F3—Si1	1.6798 (17)
C7—N3	1.478 (3)	F4—Si1	1.6868 (15)
C7—C8	1.495 (4)	F5—Si1	1.6677 (16)
C7—H7A	0.9700	F6—Si1	1.6915 (16)
C7—H7B	0.9700	F7—Si2	1.6848 (14)
C8—C9	1.507 (4)	F8—Si2	1.6858 (16)
C8—H8A	0.9700	F9—Si2	1.6741 (15)
C8—H8B	0.9700	F10—Si3	1.6848 (17)
C9—C9^i^	1.506 (5)	F11—Si3	1.6830 (14)
C9—H9A	0.9700	F12—Si3	1.6759 (14)
C9—H9B	0.9700	Si2—F9^iii^	1.6741 (15)
C10—N4	1.465 (3)	Si2—F7^iii^	1.6849 (14)
C10—C11	1.489 (4)	Si2—F8^iii^	1.6858 (16)
C10—H10A	0.9700	Si3—F12^iv^	1.6760 (14)
C10—H10B	0.9700	Si3—F11^iv^	1.6830 (14)
C11—C12	1.508 (4)	Si3—F10^iv^	1.6848 (17)
C11—H11A	0.9700		
			
N1—C1—C2	112.6 (3)	H12A—C12—H12B	107.6
N1—C1—H1A	109.1	C1—N1—H1NB	109.5
C2—C1—H1A	109.1	C1—N1—H1NA	109.5
N1—C1—H1B	109.1	H1NB—N1—H1NA	109.5
C2—C1—H1B	109.1	C1—N1—H1NC	109.5
H1A—C1—H1B	107.8	H1NB—N1—H1NC	109.5
C3—C2—C1	112.9 (3)	H1NA—N1—H1NC	109.5
C3—C2—H2A	109.0	C6—N2—H2NA	109.5
C1—C2—H2A	109.0	C6—N2—H2NC	109.5
C3—C2—H2B	109.0	H2NA—N2—H2NC	109.5
C1—C2—H2B	109.0	C6—N2—H2NB	109.5
H2A—C2—H2B	107.8	H2NA—N2—H2NB	109.5
C2—C3—C4	115.1 (3)	H2NC—N2—H2NB	109.5
C2—C3—H3A	108.5	C7—N3—H3NA	109.5
C4—C3—H3A	108.5	C7—N3—H3NB	109.5
C2—C3—H3B	108.5	H3NA—N3—H3NB	109.5
C4—C3—H3B	108.5	C7—N3—H3NC	109.5
H3A—C3—H3B	107.5	H3NA—N3—H3NC	109.5
C5—C4—C3	116.0 (3)	H3NB—N3—H3NC	109.5
C5—C4—H4A	108.3	C10—N4—H4NA	109.5
C3—C4—H4A	108.3	C10—N4—H4NB	109.5
C5—C4—H4B	108.3	H4NA—N4—H4NB	109.5
C3—C4—H4B	108.3	C10—N4—H4NC	109.5
H4A—C4—H4B	107.4	H4NA—N4—H4NC	109.5
C4—C5—C6	117.4 (3)	H4NB—N4—H4NC	109.5
C4—C5—H5A	108.0	F5—Si1—F1	91.67 (8)
C6—C5—H5A	108.0	F5—Si1—F3	91.24 (10)
C4—C5—H5B	108.0	F1—Si1—F3	91.04 (9)
C6—C5—H5B	108.0	F5—Si1—F2	179.11 (10)
H5A—C5—H5B	107.2	F1—Si1—F2	88.89 (8)
N2—C6—C5	114.0 (3)	F3—Si1—F2	89.43 (9)
N2—C6—H6A	108.8	F5—Si1—F4	89.45 (8)
C5—C6—H6A	108.8	F1—Si1—F4	178.85 (9)
N2—C6—H6B	108.8	F3—Si1—F4	89.17 (9)
C5—C6—H6B	108.8	F2—Si1—F4	89.98 (8)
H6A—C6—H6B	107.6	F5—Si1—F6	88.99 (9)
N3—C7—C8	112.4 (2)	F1—Si1—F6	89.33 (8)
N3—C7—H7A	109.1	F3—Si1—F6	179.56 (10)
C8—C7—H7A	109.1	F2—Si1—F6	90.33 (9)
N3—C7—H7B	109.1	F4—Si1—F6	90.46 (8)
C8—C7—H7B	109.1	F9^iii^—Si2—F9	180.0
H7A—C7—H7B	107.9	F9^iii^—Si2—F7	89.15 (8)
C7—C8—C9	115.8 (3)	F9—Si2—F7	90.85 (8)
C7—C8—H8A	108.3	F9^iii^—Si2—F7^iii^	90.85 (8)
C9—C8—H8A	108.3	F9—Si2—F7^iii^	89.15 (8)
C7—C8—H8B	108.3	F7—Si2—F7^iii^	180.0
C9—C8—H8B	108.3	F9^iii^—Si2—F8	90.78 (9)
H8A—C8—H8B	107.4	F9—Si2—F8	89.22 (9)
C9^i^—C9—C8	112.9 (3)	F7—Si2—F8	89.29 (8)
C9^i^—C9—H9A	109.0	F7^iii^—Si2—F8	90.71 (8)
C8—C9—H9A	109.0	F9^iii^—Si2—F8^iii^	89.22 (9)
C9^i^—C9—H9B	109.0	F9—Si2—F8^iii^	90.78 (9)
C8—C9—H9B	109.0	F7—Si2—F8^iii^	90.71 (8)
H9A—C9—H9B	107.8	F7^iii^—Si2—F8^iii^	89.29 (8)
N4—C10—C11	112.4 (2)	F8—Si2—F8^iii^	180.0
N4—C10—H10A	109.1	F12—Si3—F12^iv^	180.0
C11—C10—H10A	109.1	F12—Si3—F11^iv^	89.08 (8)
N4—C10—H10B	109.1	F12^iv^—Si3—F11^iv^	90.92 (8)
C11—C10—H10B	109.1	F12—Si3—F11	90.92 (8)
H10A—C10—H10B	107.8	F12^iv^—Si3—F11	89.08 (8)
C10—C11—C12	113.3 (3)	F11^iv^—Si3—F11	180.000 (1)
C10—C11—H11A	108.9	F12—Si3—F10^iv^	89.24 (9)
C12—C11—H11A	108.9	F12^iv^—Si3—F10^iv^	90.76 (9)
C10—C11—H11B	108.9	F11^iv^—Si3—F10^iv^	90.66 (9)
C12—C11—H11B	108.9	F11—Si3—F10^iv^	89.34 (9)
H11A—C11—H11B	107.7	F12—Si3—F10	90.76 (9)
C12^ii^—C12—C11	114.8 (3)	F12^iv^—Si3—F10	89.24 (9)
C12^ii^—C12—H12A	108.6	F11^iv^—Si3—F10	89.34 (9)
C11—C12—H12A	108.6	F11—Si3—F10	90.66 (9)
C12^ii^—C12—H12B	108.6	F10^iv^—Si3—F10	180.00 (12)
C11—C12—H12B	108.6		

Symmetry codes: (i) −*x*, −*y*+2, −*z*+1; (ii) −*x*, −*y*+1, −*z*; (iii) −*x*, −*y*+1, −*z*+1; (iv) −*x*, −*y*+2, −*z*.

### Hydrogen-bond geometry (Å, º)

**Table:**

*D*—H···*A*	*D*—H	H···*A*	*D*···*A*	*D*—H···*A*
N2—H2*NC*···F5	0.89	2.31	3.062 (3)	142
N2—H2*NC*···F6	0.89	2.27	3.063 (3)	148
N2—H2*NB*···F7	0.89	2.52	3.095 (3)	123
N2—H2*NB*···F8	0.89	2.03	2.917 (3)	175
N3—H3*NA*···F1	0.89	2.06	2.934 (3)	169
N4—H4*NA*···F8	0.89	2.34	3.095 (3)	143
N4—H4*NA*···F9	0.89	2.14	2.923 (3)	146
N4—H4*NC*···F6	0.89	2.00	2.885 (3)	172
N1—H1*NB*···F11^i^	0.89	2.07	2.895 (3)	155
N1—H1*NA*···F10^v^	0.89	2.01	2.869 (3)	163
N1—H1*NC*···F2^vi^	0.89	2.02	2.857 (2)	155
N2—H2*NA*···F7^vii^	0.89	2.02	2.906 (3)	170
N3—H3*NB*···F10^iv^	0.89	2.48	3.239 (3)	144
N3—H3*NB*···F12	0.89	2.13	2.907 (2)	145
N3—H3*NC*···F3^viii^	0.89	2.02	2.904 (3)	170
N3—H3*NC*···F4^viii^	0.89	2.44	3.034 (3)	124
N4—H4*NB*···F4^viii^	0.89	2.01	2.832 (3)	154
N4—H4*NB*···F5^viii^	0.89	2.51	3.086 (3)	123

Symmetry codes: (i) −*x*, −*y*+2, −*z*+1; (iv) −*x*, −*y*+2, −*z*; (v) −*x*+1, −*y*+2, −*z*+1; (vi) *x*, *y*, *z*+1; (vii) −*x*+1, −*y*+1, −*z*+1; (viii) *x*−1, *y*, *z*.
